# CpG Improves Influenza Vaccine Efficacy in Young Adult but Not Aged Mice

**DOI:** 10.1371/journal.pone.0150425

**Published:** 2016-03-02

**Authors:** Alejandro Ramirez, Mary Co, Anuja Mathew

**Affiliations:** Division of Infectious Diseases and Immunology, Department of Medicine, University of Massachusetts Medical School, Worcester, MA, United States of America; Georgia State University, UNITED STATES

## Abstract

Several studies have shown a reduced efficacy of influenza vaccines in the elderly compared to young adults. In this study, we evaluated the immunogenicity and protective efficacy of a commercially available inactivated influenza vaccine (Fluzone^®^) in young adult and aged mice. C57/BL6 mice were administered a single or double immunization of Fluzone^®^ with or without CpG and challenged intranasally with H1N1 A/California/09 virus. A double immunization of Fluzone^®^ adjuvanted with CpG elicited the highest level of protection in young adult mice which was associated with increases in influenza specific IgG, elevated HAI titres, reduced viral titres and lung inflammation. In contrast, the vaccine schedule which provided fully protective immunity in young adult mice conferred limited protection in aged mice. Antigen presenting cells from aged mice were found to be less responsive to *in vitro* stimulation by Fluzone and CpG which may partially explain this result. Our data are supportive of studies that have shown limited effectiveness of influenza vaccines in the elderly and provide important information relevant to the design of more immunogenic vaccines in this age group.

## Introduction

Influenza A virus is a seasonal virus which affects 32,000 individuals in the US and 600,000 worldwide each year [1]. Those at most risk from complications include young children, pregnant women, asthma sufferers, immune compromised individuals and the elderly. The elderly account for up to 80% of hospitalisations and 95% of influenza associated mortality [2]. To help reduce these severe outcomes, public health authorities recommend that individuals over 65 should receive yearly influenza vaccination. However, several clinical trials and cohort studies have reported low rates of vaccine efficacy in the elderly, as judged by statistically significant reductions in influenza associated hospitalisation rates or seroconversion post vaccination [3–8].

No single factor has been identified to explain this lower vaccine efficacy in the elderly, but it is thought that various age-related deficiencies in immune responses contribute. From human and animal studies, it is known that aging is associated with decreased responses to innate immune stimuli and abnormal production of various cytokines, chemokines and growth factors. In addition, immune cells from older subjects have a number of signalling defects, slower rates of dendritic cell migration, activation and compromised antigen presentation [9–19]. Furthermore, antibody production and antibody half-life is diminished in the aged and the available T-cell repertoire is diminished due to thymic involution, as well as the number of total naive T-cell and B lymphocytes in the elderly [20–23]. All of these factors are likely to account for decreased vaccine responses in older individuals, since vaccines rely on a fully functional immune system to generate protective immune memory.

In human and murine studies, adjuvants and increased antigen doses have been used to generate better vaccine responses. Influenza vaccines administered with the squalene based M59 adjuvant generated higher seroprotection rates in people over 60 [24, 25]. The Fluzone High-Dose vaccine formulation made by Sanofi Pasteur which contains four times the active antigen content as in the conventional vaccine received by young adults, increased seroconversion rates from 30% to 50% to H1N1 in the elderly [26]. Pica et. al also showed that a higher dose of live attenuated influenza vaccine is necessary to induce protective immunity in aged mice compared to young adult mice [27]. Also in mice, administering alum or Poly I:C with an influenza vaccine substantially increased survival in older animals [28]. The use of replication incompetent vaccine vectors derived from Venezuelan Equine Encephalitis virus carrying influenza transgenes protected aged mice from lethal homologous and heterologous influenza challenge [29]. These findings indicate that despite age associated immune atrophy, it is possible to partially rescue vaccine efficacy in the elderly by augmenting the innate immune response either with higher antigen doses, co-stimulation, priming or the use of adjuvants [30].

In this study, we compared influenza vaccine responses to the commercially available 2011–2012 inactivated influenza vaccine Fluzone (FZ) and report the effects of co-administering a well characterized adjuvant CpG in young adult and aged C57BL/6J mice. We show that in this mouse model, the efficacy of Fluzone in aged mice is lower than in young adult mice and that CpG co-administration more significantly improves vaccine efficacy in young adult but not aged mice.

## Materials and Methods

### Mice

Young adult (6–8 weeks) and middle aged (8–9 months) male and female C57BL/6J were purchased from Jackson Laboratories (Bar Harbor, ME). Mice were aged in house to 18–24 months old or purchased from the National Institute of Ageing NIA. Throughout the text “young” or “young adult” refers to 6–8 week old mice and “aged/old” refers to 18–24 month old mice. Groups of 5 to 8 mice were used for each test group/condition. Ethics Statement: All experiments were performed in accordance with guidelines of the Institutional Animal Care and Use Committee of the University of Massachusetts Medical School and the recommendations in the *Guide for the Care and Use of Laboratory Animals* (Institute of Laboratory Animal Resources, National Research Council, National Academy of Sciences, 1996). The Institutional Animal Care and Use Committee at UMASS Medical School approved the animal protocol used in this study.

### Virus stocks and Infection

H1N1 A/California/09 influenza virus was obtained from Sanofi Pasteur (NYMC X-179A Lot H1SWSP 4182011–4), and propagated in MDCK cells to a 6.5 HAU/μl virus stock. Mice were inoculated intranasally with 4x10^5^ p.f.u. of influenza H1N1 virus diluted in 50μl of sterile PBS. Isofluorane was used to anaesthetize mice for intra-nasal delivery of the virus. Animals were monitored daily after H1N1 infection for survival and weight loss. Mice were bled periodically to monitor antibody production using standard facial vein bleeding protocols. Mice were sacrificed for ethical considerations when they lost >20% of their original body weight as stated in our approved protocol. For euthanasia, asphyxiation through CO_2_ was used and all efforts were made to minimize suffering. No unexpected deaths were observed during the infections. Weight loss charts show the average weight loss for 5 to 8 animals +/- standard error.

### Vaccines and Immunization

The 2011–2012 Fluzone^®^ inactivated vaccine, (Sanofi Pasteur), contains 15 μg each of HA protein from the A/California/09 H1N1, A/Victoria/09 H3N2 and B/Brisbane/08 viruses. All mice received a high vaccine dose, corresponding to a total of 1.2 μg of HA protein from each of the virus strains in the Fluzone^®^ vaccine. Mice received one of four vaccine formulations -Fluzone alone (FZ; 40μl of FZ, 60 μl sterile PBS), Fluzone + 10μg CpG (FZ+CpG; 40μl of FZ, 10μl CpG, 50 μl PBS), 10μg CpG (CpG; 10μl of CpG, 90 μl PBS) or PBS alone (100μl PBS), administered as an intra-muscular vaccination into the caudal thigh muscle. CpG was purchased from Invivogen (Cat.no. tlrl-2395 /sequence 5’-tcgtcgttttcggcgc:gcgccg-3’).

### Flow cytometry

Cell culture suspensions or single cell organ homogenates were washed in RPMI/10% FBS first followed by FACS Buffer (PBS/2% FBS/0.1% sodium azide), then incubated with a titrated amount of these antibodies: CD3 FITC, CD11b AlexaFluor 700, CD11c APC, MHCII PECy5, F4/80 Pacific Blue, CD80 PerCP Cy5.5, Ly6C FITC, CD37APCCy7, CD44PerCP, TNF-α PECy7 (BD BioSciences or eBioscience, San Diego, CA) at 4°C for an additional 30 minutes. Cells were fixed with BD Stabilizing Fixative^™^ (BD Biosciences) for analysis. Data were collected on a BD FACSAria^™^ equipped with Diva v7.0 and CS&T v2.0 software (BD Biosciences) and analyzed using FlowJo version 10.

### Plaque Assay

Lung homogenates were freeze-thawed three times, centrifuged at 4000 g and supernatants titrated in doubling dilutions on Madine Darby canine kidney (MDCK) cell monolayers in flat bottomed 96 well plates. After incubation at 37°C for 3 hours, samples were over-laid with 1% methylcellulose and incubated for 72 hours at 37°C. Cell monolayers were fixed and washed, then stained with Crystal Violet solution to visualize plaques. Infectious units were then enumerated by light microscopy and total plaque forming units per lung quantified (number of plaques × dilution factor × total volume of lung homogenate).

### Cell Culture and cytokine detection

Bone marrow derived macrophages (BMM) and dendritic cells (BMDC) were generated as described previously [31]. Briefly, femoral bone was harvested from either young adult (6–8 weeks) or aged (18–24 months) mice and washed in ethanol. The medullar pulp was washed out in sterile RPMI medium. RBC were lysed and after washing, the cell suspension was split into 2 plates. One plate was differentiated into macrophages (F4/80+CD11b+) by culturing with L929 cell line conditioned media for 7–9 days. Dendritic cells (CD11c+/CD11b^med^/MHCII^Hi^) were induced by culturing with GM-CSF (20ng/ml) enriched RPMI/10% FBS medium for 7–9 days. For *in vitro* stimulation, 1x10^6^ cells were stimulated with 2.5μg/ml of CpG (Invivogen) and/or 2μg/ml of Fluzone for 12 hrs at 37°C. TNF-α levels in cell culture medium were detected using a commercial ELISA kit (eBioscience, San Diego, CA). The assay was run in duplicate, results are shown as mean values and are representative of 2 separate experiments.

### Anti-Influenza IgG detection and HAI assay

#### IgG detection

Microtiter plates were coated with 50HA/well UV inactivated H1N1 A/California/09 influenza overnight at 4°C. Plates were washed and then blocked with 10%BSA/PBS and sera from immune or naïve mice was added and incubated for 3 hrs at room temperature. Plates were washed and incubated with Goat anti-Mouse IgG- Peroxidase Conjugate (Millipore, Temecula, CA) for 90 min at room temperature (diluted in 5%BSA/PBS). After washing, bound antibody was detected on all plates by incubating with TMB peroxidise substrate (KPL, Gaithersburg, MD) in the dark for 20 minutes. The reaction was then stopped with 50μl 2M HCL and plates were read at 450nm with a 570nm correction. Absorbance values obtained from sera of naïve mice were subtracted from all values and converted to relative AU x10^3^.

#### HAI assay

Two fold serial dilutions (up to 12) of heat inactivated sera from vaccinated mice and controls were mixed with 4HAU of H1N1 A/California/09 virus and incubated for 30 min at room temperature. An equal volume of 1% chicken red blood cell (CRBC) suspension was added to all wells and incubated for 45 min at room temperature. All samples were run in duplicate wells on U-bottom 96 well plates. The reciprocal of the highest dilution of sera to successfully inhibit CRBC agglutination was defined as the HAI titre of the serum sample.

### Statistics

We used Graphpad Prism software to perform non-parametric statistical analysis across no more than 2 groups of interest. Significance* was set as P<0.05

## Results

### CpG adjuvant improves efficacy of Fluzone^®^ vaccine to H1N1 challenge in young adult mice

**Fig 1:**
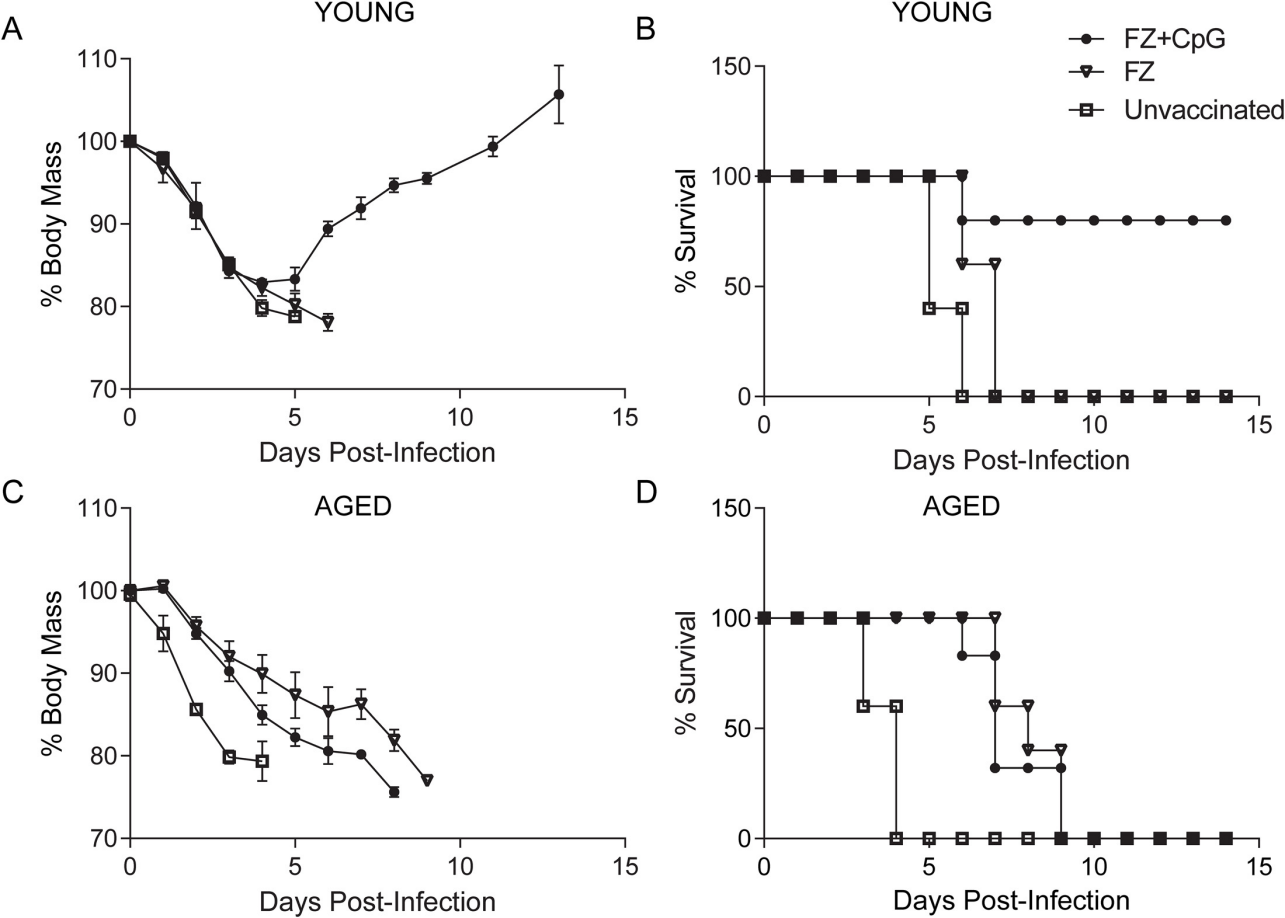
Single Fluzone vaccination with or without adjuvants is non-protective in aged mice. Young adult (6–8 weeks) and aged (18–24 months) C57/BL6 mice (n = 5–8 mice per group) were vaccinated with Fluzone (FZ), Fluzone+CpG (FZ+CpG) or left unvaccinated as controls. Approximately 4 weeks later, all groups were challenged with 1x10^4^ p.f.u of A/California/09 H1N1 virus. Weight loss (A) and (C) and survival (B) and (D) were monitored daily in young adult and aged mice respectively. Individual data points represent average weight loss within the group of surviving mice +/-SEM.

A group of young adult C57BL/6 mice (6–8 weeks; n = 5–8) was vaccinated with a commercial influenza vaccine, Fluzone (FZ). Analysis of serum 4 weeks post vaccination showed that H1N1 specific IgG was detectable; however, no hemagglutination inhibition activity (HAI <5) was observed (Table 1). When challenged with H1N1 A/California/09 influenza virus, these mice experienced weight loss similar to unvaccinated controls (Fig 1A) and 100% mortality (Fig 1B). We wanted to investigate whether a well characterized innate immune TLR9 agonist (CpG) could improve the immunogenicity and the efficacy of FZ in this model. Four weeks after immunization, young adult mice immunized with FZ+CpG had H1N1 specific IgG titres over 3 times greater compared to FZ alone (Table 1) and had detectable HAI activity equivalent to a 16 fold increase post vaccination (HAI = 80) (Table 1). This increased serum IgG and HAI activity was associated with a decrease in peak weight loss following infection followed by full recovery (Fig 1A), with improved clinical scores as judged by increased activity and less fur ruffling (data not shown). Furthermore, mortality in the unvaccinated and unadjuvanted (FZ) groups reached 100% following infection compared to only 20% in the adjuvanted (FZ+CpG) group (Fig 1B).

**Table 1:** Heme agglutination inhibition and Influenza-specific IgG titers in sera of immunized mice.

		Young Adult			Aged		
		Pre Vaccine	Post Vaccine	Post Boost	Pre Vaccine	Post Vaccine	Post Boost
FZ	IgG	0	1100		0	3100	
	HAI	<5	<5		<5	<5	
FZ+CpG	IgG	0	3800		0	2600	
	HAI	<5	80		<5	<5	
FZ x2	IgG	0	1900	3600	n.d	n.d.	n.d.
	HAI	<5	20	40			
(FZ+CpG) x2	IgG	0	3400	3800	0	4600	3400
	HAI	<5	40	80	<5	<5	<5

Sera from pooled mice (n = 5 per group) were used to assess Influenza-specific IgG and HAI titers.

Equal volumes of serum from 5 mice were pooled and mean values of duplicate wells are shown. ELISA data are representative of two separate experiments.

n.d. = not determined

FZ = Fluzone.

### CpG adjuvant fails to improve efficacy of Fluzone^®^ vaccine to H1N1 challenge in aged mice

Similar to young adult mice, aged mice (18–24 months; n = 5–8) vaccinated with FZ had positive anti-H1N1 IgG titres 4 weeks after immunization compared to unvaccinated mice, but no detectable HAI activity (Table 1). However, unlike young adult mice, the addition of a CpG adjuvant (FZ+CpG) did not show either an increase in Influenza A virus (IAV) specific IgG titre or HAI activity when compared to FZ alone (Table 1). Vaccinated aged mice did show slightly lower weight loss at each time point compared to unvaccinated controls following influenza infection (Fig 1C). Despite this, both vaccinated and unvaccinated aged mice succumbed to H1N1 infection with 100% mortality in those groups (Fig 1D). Since the adjuvanted vaccine was partially protective in young adult mice but ineffective at preventing influenza mortality in aged mice, it confirmed that age related differences in influenza vaccination efficacy seen in young and aged human subjects could be modeled in mice.

### APC from aged mice are less responsive to Fluzone with CpG adjuvant

We observed that the addition of CpG increased the efficacy of FZ in young but not aged mice. CpG has been shown to act as an adjuvant by stimulating innate responses in antigen presenting cells (APC) which first encounter the vaccine. Previous work has already shown that TNF-α release is compromised *in vitro* and in response to influenza virus infection in aged mice [32, 33]. Similarly MHC class II expression is lower in response to IAV, in cells from older adults [34]. We stimulated bone marrow derived macrophages (BMM) and dendritic cells (BMDC) from both young adult and aged mice *in vitro* with FZ or FZ+CpG and examined TNF-α production and MHC class II cell surface expression (S1 Fig).

**Fig 2:**
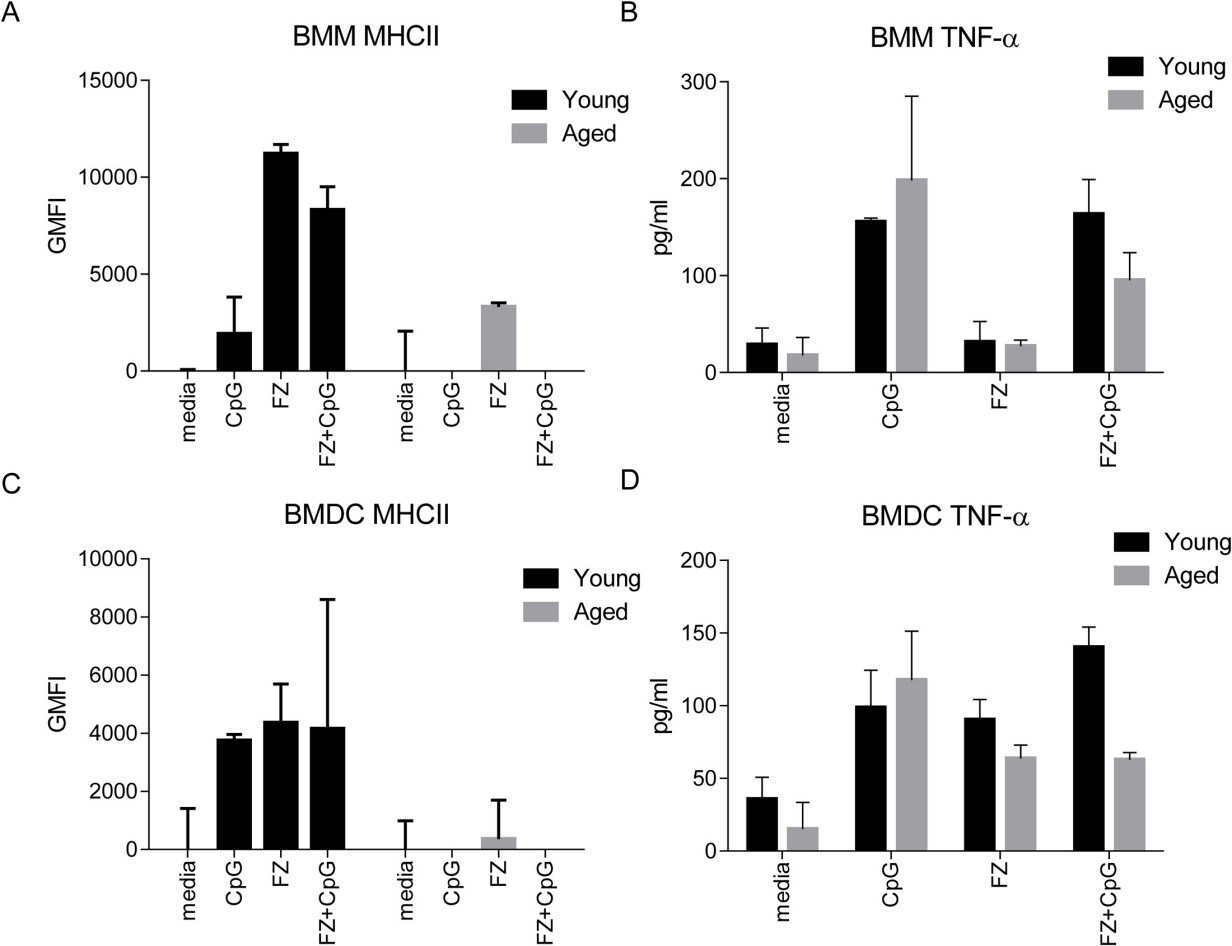
Antigen presenting cells from aged mice do not upregulate MHC class II expression in response to vaccine components *in vitro*. Bone marrow derived macrophages (BMM) or dendritic cells (BMDC) from young adult (6–8 weeks, dark bars) and aged (18–24 months, light bars) were stimulated *in vitro* with CpG, Fluzone (FZ) or both (FZ+CpG) overnight. Up regulation of MHC class II relative to unstimulated cells (media) was measured using flow cytometry on (A) BMM (F4/80+/CD11b+) and (C) BMDC (CD11c+/CD11b^med^ /F4/80-). TNF-α production was measured by ELISA in supernatants from (B) BMM and (D) BMDC. Readings were taken as an average of triplicate wells. Results shown are mean values + S.D and are representative of 2 independent experiments.

BMM from young adult mice upregulated MHC class II in response to FZ and FZ+CpG with minimal up regulation to CpG alone (Fig 2A). TNF-α levels were low when stimulated with FZ alone but were significantly boosted in the presence of CpG (Fig 2B). By contrast, BMM from aged mice had comparatively low MHC class II up regulation in response to FZ, and no detectable up regulation in the presence of CpG (Fig 2A). Interestingly TNF-α production was similar in BMM from young adult and aged mice (Fig 2B). BMDCs from young adult mice were also responsive to activation by FZ, CpG alone and FZ+CpG, showing strong up regulation of MHC class II and TNF-α release (Fig 2C and 2D). The highest TNF-α concentration was measured in cells stimulated with FZ+CpG (Fig 2D). There was minimal MHCII up regulation by BMDC derived from aged mice (Fig 2C). Additionally there was a significant reduction in TNF-α in the FZ+CpG samples compared to BMDC from young adult mice (Fig 2D). Taken together, we found that APCs derived from aged mice were less activated when stimulated *in vitro* with either FZ or FZ+CpG, compared to cells from young adult mice.

### Repeated vaccination with CpG adjuvanted FZ provides fully protective immunity in young adult mice but limited protection in aged mice

**Fig 3:**
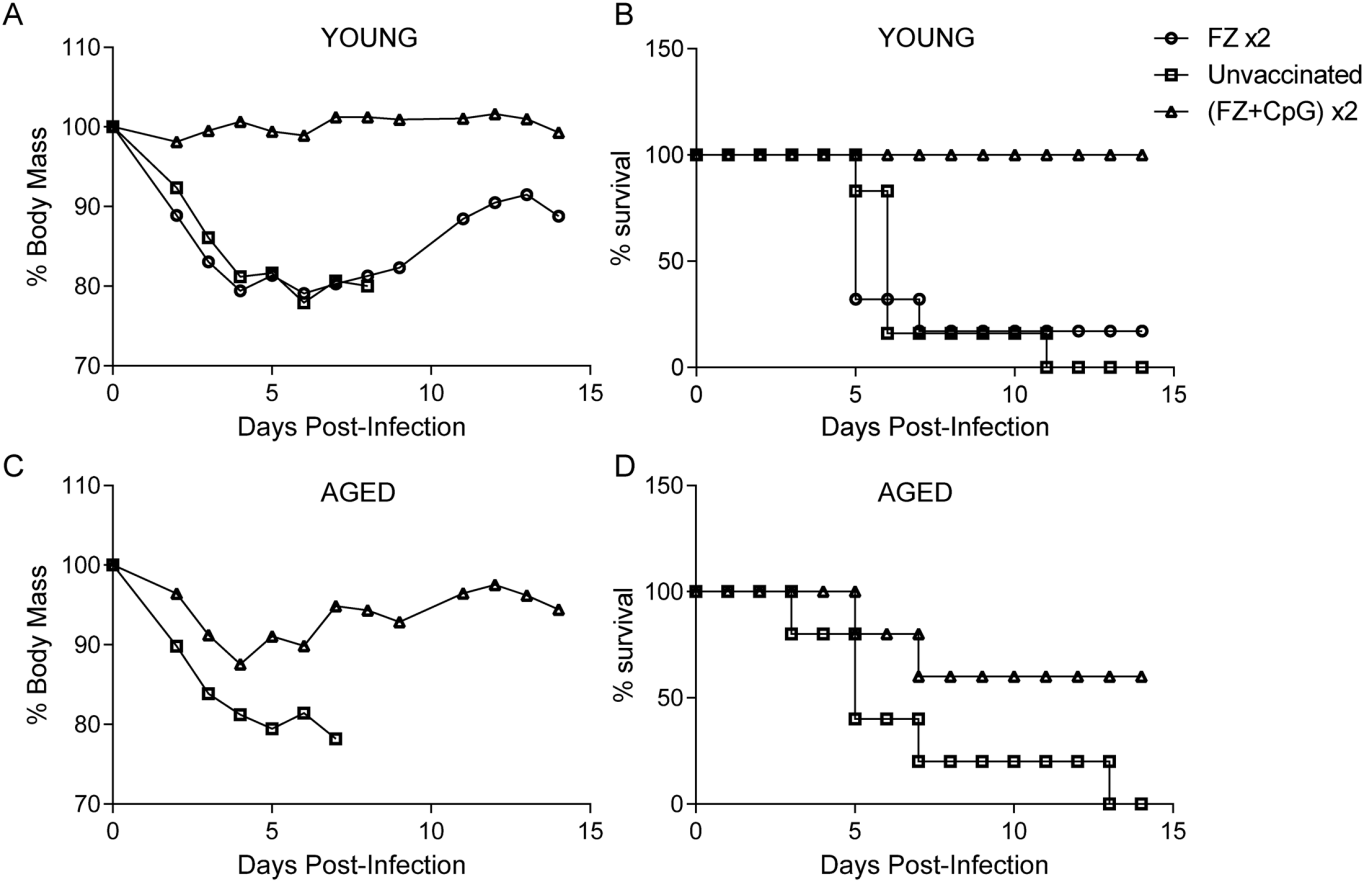
Immunization with two doses of adjuvanted Fluzone vaccine is partially protective in aged mice. Young adult (6–8 weeks) and aged (18–24 months) (n = 5-8/group) mice were vaccinated twice, 2 weeks apart, with Fluzone (FZ x2), Fluzone+CpG (FZ+CpG) x2 or left unvaccinated as controls. Approximately 4 weeks later, all groups were challenged with 1x10^4^ p.f.u of A/California/09 H1N1virus. (A) and (C) Weight loss and (B) and (D) survival were monitored daily in young adult and aged mice. Individual data points represent average weight loss within the group of surviving mice +/-SEM.

In previous experiments, we demonstrated that the co-administration of CpG+FZ improved antibody responses and protection from H1N1 IAV in young adult but not aged mice. We wanted to investigate whether the addition of a second vaccination would add further benefit in young adult mice and lead to some protection in aged mice. Young adult mice were vaccinated with FZ alone or FZ+CpG at 6 weeks and 2 weeks before challenge with A/California/09 H1N1 virus. Our data indicate after a double CpG adjuvanted immunization (FZ+CpG) x2, young adult mice were fully protected from H1N1 IAV associated weight loss and mortality (Fig 3A and 3B). Conversely, the group that received two immunizations with FZ alone still experienced substantial weight loss and 80% group mortality. Four weeks after primary immunization, young adult mice in the FZ+CpG group had significantly higher levels of anti-H1N1 IgG compared to FZ alone, which is consistent with our findings. After the second immunization, both groups, (FZ+CpG) x2 and FZ x2, had comparable levels of IAV-specific IgG. However, HAI antibody levels were two times higher in the adjuvanted vs. the non-adjuvanted group (40 vs. 20) (Table 1).

We next wanted to determine if some of the benefits related to the immunization with a second dose in young adult mice could be seen in aged mice. In two separate experiments, aged mice were vaccinated at -6 and -2 weeks prior to challenge with A/California/09 H1N1virus in an identical manner to young adult mice. As the number aged mice available to us was limited, we compared only the most protective vaccine schedule (FZ+CpG *2) in young adult and aged mice. Fig 3C shows that some aged mice within the vaccinated groups showed a decrease in body mass loss following A/California/09 H1N1 infection, resulting in reduced weight loss and recovery thereafter. However, all mice showed some degree of weight loss and the overall group survival was only 60% (Fig 3D). Thus, the same vaccine formulation and immunization schedule which resulted in symptomless (asymptomatic) infection in young adult mice gave a more modest level of protection in aged mice. This benefit may have been due to cell mediated responses induced by vaccination since no HAI activity was detected.

### Protection in immunized young adult mice is associated with improved cellular immunity and reduced viral titers

**Fig 4:**
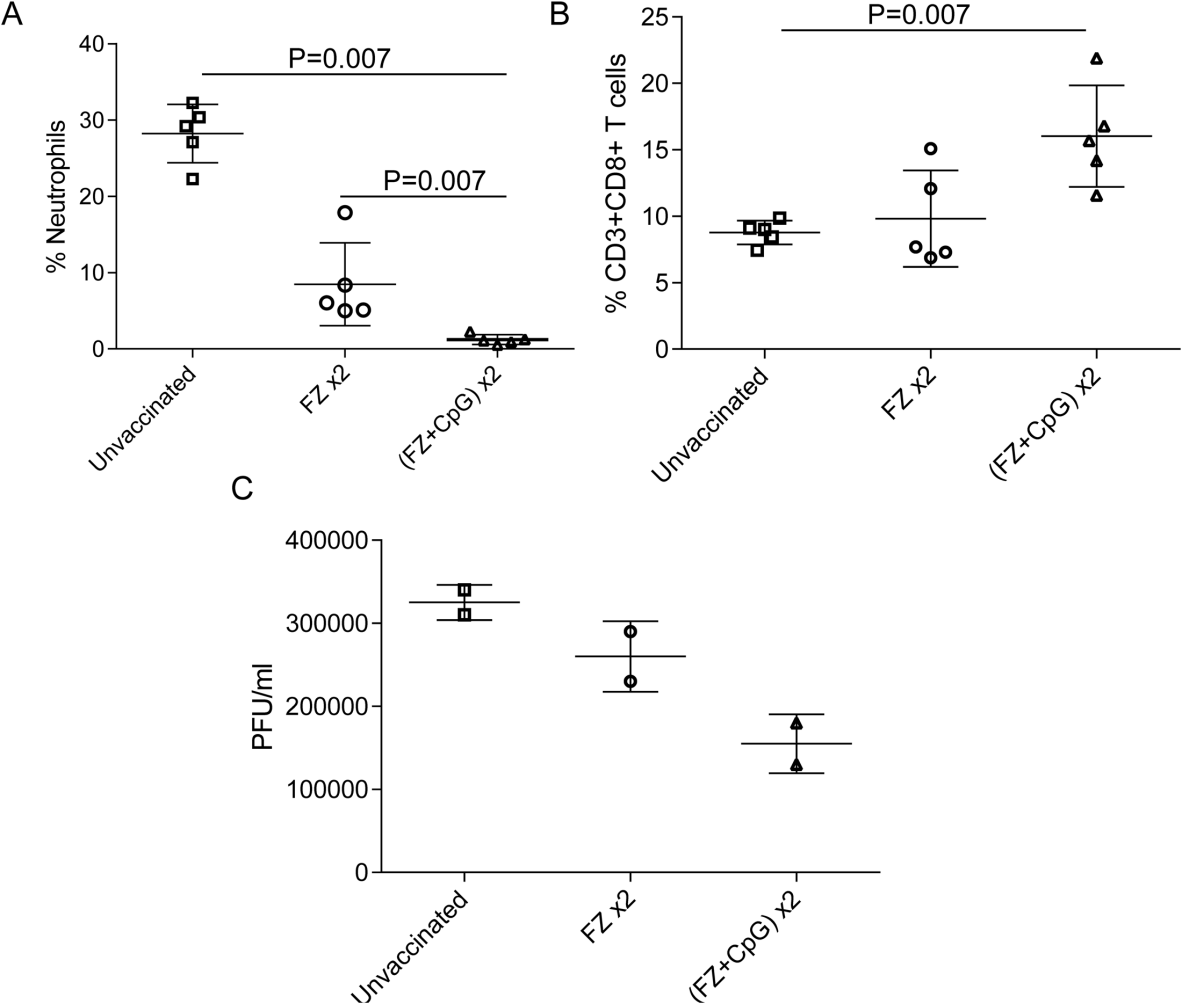
Protection in immunized young adult mice is associated with improved cellular immunity and reduced viral titers. Young adult mice (6–8 weeks) vaccinated twice, 2 weeks apart, with Fluzone (FZ x2), Fluzone+CpG (FZ+CpG) and unvaccinated controls were challenged with 1x10^4^ p.f.u of A/California/09 H1N1. Five mice per group were sacrificed at day 7 post-infection. (A) The frequency of neutrophils (CD3-/Ly6C+) and (B) CD3+/CD8+ T-cells was measured by flow cytometry in homogenates of the right lobe of the lung. Dots represent individual mice and horizontal lines represent average +/-SEM where *p<0.05. (C) Lung virus titers from pooled mice (n = 5 per group) were determined day 7 post challenge using homogenates from the left lobe of the lung. Equal volumes of lung homogenate were pooled from 5 mice in each group and the horizontal bar represents average of duplicate wells. Data shown is representative of 2 independent experiments.

One of the main causes of influenza associated morbidity is immunopathology mediated by a large influx of neutrophils in the late stages of viral clearance [35]. Fig 4A shows that young adult mice immunized twice with FZ+CpG have a reduced influx of neutrophils present in the lungs at day 7 post infection compared to unvaccinated mice and mice vaccinated twice with FZ alone (p<0.007). A reduction in lung neutrophilia is generally a sign of reduced local inflammation and immunopathology. Both vaccinated groups had a higher percentage of CD4+ cells present in the lung after infection, relative to unvaccinated controls (not shown). However only mice immunized twice with FZ+CpG had a significantly higher percentage of CD8+ T-cells in the lung following infection (Fig 4B). Finally, we assessed viral burden in the lungs and found that mice immunized twice with FZ+CpG had lower viral titres compared to mice immunized twice with FZ and the unvaccinated controls (Fig 4C).

## Discussion

Several studies have demonstrated limited efficacy of inactivated influenza vaccine in elderly individuals compared to younger adults. Although reduced vaccine efficacy in the elderly is generally attributed to immunosenescence, the mechanisms leading to this phenomenon are not fully understood [36]. In this study we used a mouse model to analyze the disparity in efficacy of a split virus inactivated influenza vaccine in young adult and aged mice. Furthermore, we investigated whether vaccine efficacy could be improved in aged mice by co-administration of a CpG adjuvant.

Young adult mice vaccinated with Fluzone and CpG prior to an IAV challenge showed a clear benefit in regards to clinical symptoms (activity, fur ruffling and weight loss) and mortality compared to unvaccinated mice. Young adult mice were asymptomatic and protected to an H1N1 live-virus challenge after a second dose of Fluzone and CpG which were associated with increases in influenza specific IgG, elevated HAI titres, reduced viral titres and lung inflammation. In contrast, the benefits were more modest in aged mice. All immunization protocols with or without adjuvant resulted in poorer protection in aged compared to young adult mice. Strikingly, the same immunization schedule (FZ+CpG) x2 doses which conferred full protection in young adult mice resulted in only partial protection (60% survival) and no HAI activity in aged mice. APC from older mice showed diminished responses to FZ alone and to FZ+CpG, which may in part account for the low vaccine efficacy. It is possible that aged mice have a higher susceptibility to IAV than young mice, but it would be difficult to extricate increased susceptibility from a diminished immune response. Other groups have found similar low responses to purified HA and CpG in aging humans [37, 38]. Interestingly, we found that following adjuvanted vaccination, young adult and aged mice develop similar amounts of anti-influenza specific IgG in the serum, but only sera from young adult mice tested positive in HAI assays, suggesting that perhaps antibody quality may be compromised in older mice. Studies have reported that antibodies from aged individuals have a shorter lifespan and lower affinities [3, 39].

Due to limited sera from aged mice we did not assess influenza-specific IgG isotypes and other functions of antibodies (ADCC, CDL etc) in sera from vaccinated young adult and aged mice. In future studies it would be desirable to expand the panel to other antibody isotypes and functions, which may reveal age associated differences in influenza-specific IgG functions. Though results were more striking in young adult mice, our data did show a modest benefit from a two dose immunization regimen and the use of an adjuvanted influenza vaccine in aged mice. Our results are consistent with medium sized trials which have shown that an MF59 adjuvanted influenza vaccine (Fluad) had much stronger immunogenicity in patients over 65 years old, generating higher anti-IAV titres post vaccination and faster seroconversion times [40, 41]. Similarly MF59 adjuvanted Fluad induced higher HAI titres and longer antibody persistence in infants and young children [42].

It is becoming clear that vaccination strategies which are designed, tested and approved in young adult cohorts may not have comparable results in the elderly [6, 43]. Approved inactivated influenza vaccines in the US do not contain adjuvants, but data from our studies and others suggest that these may be required to achieve adequate protection levels in the elderly. In addition, in the context of new pandemic strains to which there is no prior immunity or cross-reactivity, full protection may require double immunization, the addition of vaccine adjuvants or both.

## Supporting Information

S1 Fig MHC II expression in BMM following in vitro stimulation. Representative flow cytometry plots of MHC II expression in BMM (F4/80+/CD11b+) from aged mice following no treatment (media) or treatment with FZ for 12 hours. BMM have moderate upregulation of MHC II expression following treatment with FZ compared to unstimulated cells.
(TIF)
